# Two Subpopulations of Human Monocytes That Differ by Mitochondrial Membrane Potential

**DOI:** 10.3390/biomedicines9020153

**Published:** 2021-02-04

**Authors:** Nikita G. Nikiforov, Anastasia Ryabova, Marina V. Kubekina, Igor D. Romanishkin, Kirill A. Trofimov, Yegor S. Chegodaev, Ekaterina Ivanova, Alexander N. Orekhov

**Affiliations:** 1 Laboratory of Medical Genetics, Institute of Experimental Cardiology, National Medical Research Center of Cardiology, 121552 Moscow, Russia; nikiforov.mipt@googlemail.com; 2 Centre of Collective Usage, Institute of Gene Biology, Russian Academy of Sciences, 125315 Moscow, Russia; marykumy@gmail.com (M.V.K.); egozavr-ch@mail.ru (Y.S.C.); 3 Prokhorov General Physics Institute, Russian Academy of Sciences, 119991 Moscow, Russia; nastya.ryabova@gmail.com (A.R.); igor.romanishkin@gmail.com (I.D.R.); 4 Yandex, 119021 Moscow, Russia; antikov@yandex.ru; 5 Engelhardt Institute of Molecular Biology, Russian Academy of Sciences, 119991 Moscow, Russia; 6 Institute of Atherosclerosis Research, Skolkovo Innovative Center, 121609 Moscow, Russia; 7 Laboratory of Angiopathology, Institute of General Pathology and Pathophysiology, 125315 Moscow, Russia; 8 Laboratory of Infection Pathology and Molecular Microecology, Institute of Human Morphology, 117418 Moscow, Russia

**Keywords:** mitochondrial membrane potential, monocyte, inflammation, MitoTracker Orange CMTMRos, atherosclerosis

## Abstract

Atherosclerosis is associated with a chronic local inflammatory process in the arterial wall. Our previous studies have demonstrated the altered proinflammatory activity of circulating monocytes in patients with atherosclerosis. Moreover, atherosclerosis progression and monocyte proinflammatory activity were associated with mitochondrial DNA (mtDNA) mutations in circulating monocytes. The role of mitochondria in the immune system cells is currently well recognized. They can act as immunomodulators by releasing molecules associated with bacterial infection. We hypothesized that atherosclerosis can be associated with changes in the mitochondrial function of circulating monocytes. To test this hypothesis, we performed live staining of the mitochondria of CD14+ monocytes from healthy donors and atherosclerosis patients with MitoTracker Orange CMTMRos dye, which is sensitive to mitochondrial membrane potential. The intensity of such staining reflects mitochondrial functional activity. We found that parts of monocytes in the primary culture were characterized by low MitoTracker staining (MitoTracker-low monocytes). Such cells were morphologically similar to cells with normal staining and able to metabolize 5-aminolevulinic acid and accumulate the heme precursor protoporphyrin IX (PplX), indicative of partially preserved mitochondrial function. We assessed the proportion of MitoTracker-low monocytes in the primary culture for each study subject and compared the results with other parameters, such as monocyte ability to lipopolysaccharide (LPS)-induced proinflammatory activation and the intima-media thickness of carotid arteries. We found that the proportion of MitoTracker-low monocytes was associated with the presence of atherosclerotic plaques. An increased number of such monocytes in the primary culture was associated with a reduced proinflammatory activation ability of cells. The obtained results indicate the presence of circulating monocytes with mitochondrial dysfunction and the association of such cells with chronic inflammation and atherosclerosis development.

## 1. Introduction

According to the current understanding, atherosclerosis is associated with local chronic inflammation in the arterial wall. The role of monocytes/macrophages, key components of the innate immune system, in atherosclerosis development is currently well understood. However, the origin of macrophages populating the atherosclerotic plaque remains a matter of debate. These cells may be resident macrophages of the arterial wall or continuously recruited from the bloodstream and differentiated. In recent years, many researchers have recognized the primary role of monocytes/macrophages in local inflammation development in the arterial wall [1]. Despite the numerous studies of activated macrophages from atherosclerotic lesions, mechanisms of inflammation resolution in lesions remain to be understood. It is possible that the chronification of inflammation is dependent on macrophage precursors—circulating blood monocytes.

The two triggering factors of the innate immune response are pathogen-associated molecular patterns (PAMPs) and damage-associated molecular patterns (DAMPs). The former comprise exogenous molecules of bacterial origin that are not present in host cells, such as lipopolysaccharides (LPSs), which are components of the outer membranes of Gram-negative bacteria. The latter have an endogenous origin but, under normal circumstances, are confined in certain cellular compartments, such as mitochondria. Mitochondria retain some features of the precursor prokaryotic organisms, including autonomous replication, the presence of prokaryotic phospholipids in the inner membrane (such as cardiolipin), and circular mitochondrial DNA (mtDNA). Despite the features that make the mitochondria resemble bacterial cells, under normal conditions, they are not recognized as pathogens by the host cell. However, under stress conditions, mitochondria can induce a proinflammatory response from host immune cells through the activation of the major signaling pathways of innate immunity: toll-like receptors (TLRs), cytosolic RNA and DNA sensors, formyl peptide receptors, and inflammasomes [2,3]. Therefore, mitochondria, besides their vital function of supplying the host cell with ATP, can act as an important component of the innate immunity modulating the proinflammatory response [4].

In our previous studies, we characterized mtDNA variants detected in circulating blood cells associated with atherosclerosis. Some of the described variants were associated with monocytes’ proinflammatory activation ability [5,6]. This allowed hypothesizing that atherosclerosis is associated with altered mitochondrial function in circulating monocytes linked to proinflammatory activity. In this work, we aimed to study the mitochondrial functional activity in circulating monocytes from healthy donors and atherosclerosis patients and reveal the possible associations between the mitochondrial function and proinflammatory activation of innate immune cells.

## 2. Materials and Methods

### 2.1. Study Participants

All study participants were free of cardiovascular disease. The extent of asymptomatic atherosclerosis was assessed using data on intima-media thickness (IMT) variability in apparently healthy individuals from the Russian population, as described previously [7]. The study protocol was approved by the Institute for Atherosclerosis Research Committee on Human Research, Moscow, and the study was conducted according to the standards of the Declaration of Helsinki. Individuals belonging to the first and the second quartiles of age-adjusted IMT distribution with no evidence of visible atherosclerotic plaques in any segment of carotid arteries were assigned to Group 1 (atherosclerosis-free individuals) (Table 1). Participants belonging to the third and the fourth quartiles of IMT distribution with no atherosclerotic plaques visualized in any segment of carotid arteries were assigned to Group 1. Participants belonging to the third and the fourth quartiles of IMT distribution with visible atherosclerotic plaques (more than 20% of the arterial lumen) in at least one segment of carotid arteries were assigned to Group 2 (subclinical/asymptomatic atherosclerosis). Participants younger than 50 years without visible atherosclerotic plaques were assigned to Group 0 (young subjects).

### 2.2. IMT and Plaque Score Evaluation

Quantitative diagnostics of atherosclerotic states was performed by high-resolution ultrasonographic measurement of the IMT of common carotid arteries. The distal segments of the right and left carotid arteries were scanned in a lateral angle. The IMT of common carotid arteries was assessed on the far wall of the distal 10 mm segment before the carotid sinus area. For atherosclerotic plaque detection, the left and right common carotid arteries, carotid sinus area, and internal and external carotid arteries were scanned in three fixed projections: anterior, lateral, and posterior. When visualizing an atherosclerotic plaque, carotid arterial stenosis was assessed in a transverse projection. Measurements of IMT and plaque stenosis were made with the M’Ath computer software (Metris, SRL, France). The average of two measurements (from the right and left arteries in a lateral position) was considered an integral indicator of the mean IMT. The following plaque score was used in this study: 0 = absence of plaques, 1 = stenosis up to 20%, 2 = stenosis 20–50%, and 3 = stenosis of >50%. Stenosis of the carotid artery lumen of more than 20% was considered as defined atherosclerotic plaque.

The baseline characteristics of the study participants are presented in Table 1. Individuals free from atherosclerosis and patients with subclinical atherosclerosis older than 50 years differed by age (59 ± 2 vs. 67 ± 2 years of age, respectively), IMT (0.71 ± 0.02 vs. 0.84 ± 0.03 mm, respectively), and plaque score (0.6 ± 0.1 vs. 2 ± 0.1, respectively) (Table 1).

### 2.3. Cell Culture

Monocytes were isolated from peripheral blood by density gradient centrifugation (ficoll, PanEco), which allowed leukocyte isolation. The collected cells were subjected to magnetic separation of CD14+ cells (Miltenyi Biotec). The obtained monocytes were cultured in 24-well culture plates in serum-free medium X-Vivo (Lonza) at a density of 106 cells/mL at 37 °C under 5% CO_2_. To assess the purity of the obtained monocyte population, cells were stained with anti-CD14-conjugated antibodies and analyzed by flow cytometry. The percentage of CD14-positive cells was over 95%. Cell viability was evaluated using Trypan Blue staining and was over 98% in all experiments. To induce proinflammatory activation, cells were incubated with 1 ug/mL of LPS (Sigma) for 24 h. Culture media samples were collected for TNF detection by means of ELISA (R&D Systems).

### 2.4. Assessment of Mitochondrial Membrane Potential (MMP) with MitoTracker Orange CMTMRos

Isolated peripheral blood CD14+ monocytes were plated in 24-well cell culture plates (106 cells/mL) in X-Vivo 10 medium, containing sterile coverslips for subsequent microscopic examination on the bottom. Cells were incubated with 100 nM of MitoTracker Orange CMTMRos (Molecular Probes) for 20 min, after which the coverslips were examined under the LSM-710 laser scanning microscope (Zeiss, Germany). The wavelength ranges used were 561 nm (excitation) and 580–620 nm (emission), and a visible-light channel was used to assess the cellular morphology. Imaging parameters (laser power and signal amplification) were adjusted prior to the experiments by using cells with ablated mitochondrial potential after treatment with 5 µM carbonyl cyanide 4-(trifluoromethoxy)phenylhydrazone (FCCP) for 30 min (Appendix B).

### 2.5. Assessment of Mitochondrial Functionality in CD14+ Human Monocytes with Low MMP

In parallel to the MitoTracker staining of living cells, CD14+ monocytes isolated from the peripheral blood of the study participants were incubated with 100 mg/L of 5-aminolevulinic acid (5-ALA) (Sigma-Aldrich, St. Louis, MO, USA) for 4 h at 37 °C in 5% CO_2_. Thirty minutes prior to the microscopic examination, 100 nM of MitoTracker Orange CMTMRos was added to the cells. Images were taken under the LSM-710 laser scanning microscope (Zeiss, Germany), equipped with a 20× Plan-Apochromat NA 0.8 objective. Cells were placed in a glass-bottom plate for microscopy. The fluorescent signals of the MitoTracker dye were recorded at wavelengths of 561 nm (excitation) and 580–620 nm (emission). The fluorescent signals of 5-ALA-induced protoporphyrin IX (PpIX) were recorded at the wavelengths of 633 nm (excitation) and 650–720 nm (emission). As a result, an overlay of the transmitted light image with fluorescent images of the MitoTracker distribution and PpIX was obtained.

### 2.6. Image Analysis

For each study participant, at least 27 images of the monocyte primary culture were obtained (each image being 225 × 225 µm^2^ in size). In total, the characteristics of at least 600 individual monocytes were assessed for each study participant.

Images were obtained using a laser scanning microscope in the Tile Scan mode. Images were separated into individual tiles and saved into a 16-bit png format, where a red pseudocolor encoded for MitoTracker fluorescence, a green pseudocolor for PpIX fluorescence, and a blue pseudocolor for the transmitted light image. The files were then processed using the ilastik software [8].

The transmitted light channel was used for pixel and object classification in the ilastik software. Pixels were classified as either an object or background using edge detection. Identified objects were classified into either singlet (single cells), clumps (large aggregates consisting of several cells), or debris (small fragments), according to their size. After that, only singlet objects with a probability of over 50% were considered. Singlets that were on the very edge of images were also discarded. The identified objects were used to calculate MitoTracker and PpIX fluorescence values for every cell. The results were then exported as a data table containing information on the patient number, object size, total and average-per-pixel PpIX, and MitoTracker fluorescence.

Statistical analysis was performed using SPSS Statistics 21 (IBM).

## 3. Results

### 3.1. Evaluation of Mitochondrial Membrane Potential in CD14+ Monocytes

**Figure 1:**
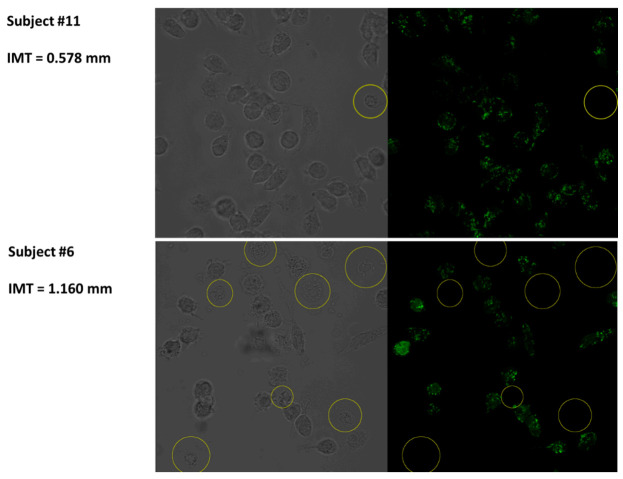
Identification of MitoTracker-low CD14+ human monocytes. Shown is a fluorescent micrograph of the monocyte primary culture from two subjects with different intima-media thickness (IMT) values, corresponding to a normal (Subject 11) and an atherosclerotic (Subject 6) status, stained with MitoTracker Orange CMTMRos. Monocytes were incubated with 100 nM of MitoTracker Orange CMTMRos for 20 min. In the left panel, the cell morphology can be seen, and the fluorescent signal from the corresponding cells is shown in the right panel. MitoTracker-low cells are indicated with yellow circles. Shown is a representative example of a field of view.

Monocytes were isolated from the peripheral blood of healthy donors and atherosclerosis patients and stained with MitoTracker Orange CMTMRos to assess mitochondrial functional activity. MitoTracker Orange CMTMRos is a cell-permeate dye that accumulates in active mitochondria with an intact membrane potential, and the staining intensity is potential dependent. The impairment of mitochondrial membrane potential results in the weakening or disappearance of MitoTracker Orange CMTMRos staining. The primary culture of human monocytes contained a mixture of cells with a normal or impaired intensity of MitoTracker Orange CMTMRos staining (Figure 1).

All primary cultures obtained from the study subjects contained MitoTracker-low cells. In such cells, a low intensity of MitoTracker Orange CMTMRos fluorescence was seen in all optic slices. Therefore, human monocytes in the primary culture differed by MitoTracker staining intensity, with some cells having a very low intensity level. These cells, however, were not dead because the cell population, as revealed by Trypan Blue staining, contained no less than 99% of live cells. It is possible that MitoTracker-low cells were in a preapoptotic state, which is characterized by morphological features such as a reduced cell size and increased granularity [9]. To investigate that possibility, we further studied the morphological features of such cells.

### 3.2. Comparative Study of the Size and Granularity of CD14+ Human Monocytes with Different MitoTracker Staining Intensities

We evaluated the possible changes in the morphology of human monocytes having different MitoTracker staining intensities in order to understand: (1) whether MitoTracker-low cells with impaired mitochondrial potential belong to a distinct population by their morphology and (2) whether or not these cells present with signs of apoptosis. We measured the cells’ surface and granularity to characterize the morphological characteristics of the monocyte population using the following assumptions. The existence of two or more morphological clusters of cells (characterized by size and granularity) indicated morphological heterogeneity of the monocyte population in the primary culture. The presence of a cluster characterized by small size and high granularity indicated a significant proportion of apoptotic cells present in the primary culture. Only one homogeneous cluster of cells with a similar morphological type present indicated the homogeneity of the cell population and a low proportion of apoptotic cells.

**Figure 2:**
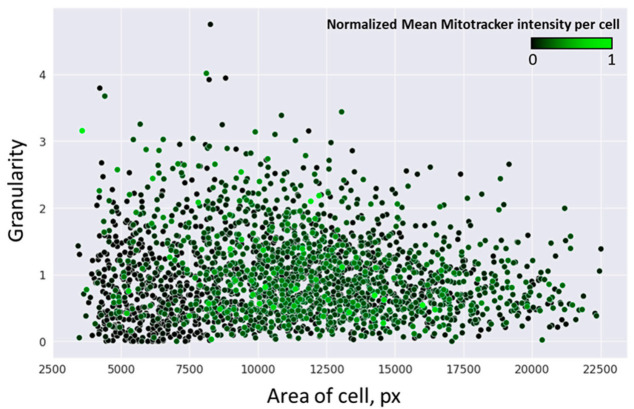
Study of the morphological heterogeneity of human monocytes varying with MitoTracker Orange CMTMRos staining intensity. Each point corresponds to one cell. Values are measured in conventional units and visualized using the CellProfiler software.

To assess the cell size and granularity, microscopic images of the cell population were examined using a machine-learning approach. Microscopic images were obtained for human monocytes in the primary culture incubated with MitoTracker Orange CMTMRos (100 nM) for 20 min. The results are presented in Figure 2.

In Figure 2, each point corresponds to one cell. The cell area (on the *x*-axis) was plotted against the cell granularity (*y*-axis) and assessed in the CellProfiler software. The intensity of the green color represents the MitoTracker staining intensity in each cell. As demonstrated on the graph, monocytes in the primary culture formed a homogeneous population, without visible separation into two or more distinct clusters. Cell size was distributed between 5000 and 20,000 conventional units, which corresponded to a variation between small and large cells by about two-fold. Such a size variation appears to be small since cultured cells are able to spread on the substrate, acquiring an irregular shape. The observed distribution of cells by size and granularity allows concluding that the monocyte primary culture was morphologically homogeneous. Within the cell population, cells with a high MitoTracker staining intensity appeared to be shifted to the right from the distribution center, whereas cells with a low staining intensity were primarily located on the left side, indicating that they had a smaller size on average. However, this shift in distribution does not allow distinguishing two cell populations characterized by high and low MitoTracker staining intensities. The obtained results also indicate that cells with a low MitoTracker staining intensity were not apoptotic since the cell granularity appeared to be homogeneous. Therefore, monocytes with high and low mitochondrial membrane potentials (MMPs), as revealed by the MitoTracker staining intensity, did not differ by size and granularity from the rest of the cell population and appeared to not be apoptotic.

Low MMP does not provide complete information on the extent and nature of mitochondrial dysfunction. Besides ATP production, mitochondria execute numerous chemical reactions, the study of which can provide more information on mitochondrial function and structural integrity. We next tested the ability of MitoTracker-low cells to heme synthesis reactions.

### 3.3. Study of Mitochondrial Function in MitoTracker-Low CD14+ Cells

To assess the ability of MitoTracker-low monocytes (with impaired MMP) to perform vital metabolic reactions specific to mitochondria, we used the approach based on culturing the cells with 5-aminolevulinic acid (ALA) followed by protoporphyrin IX (PpIX) measurement. Endogenous 5-ALA is metabolized in the cytoplasm to coproporphyrinogen III, which is transported to the mitochondria, where it is transformed to PpIX, a heme precursor [10]. The exposure of a macrophage culture to high concentrations of 5-ALA leads to excessive PpIX formation, which is either retained in the mitochondria or distributed in the cytoplasm [10]. PpIX is autofluorescent, which allows its detection in cells. We assessed the 5-ALA-induced accumulation of PpIX in cultured monocytes with low MMP to test their ability to perform mitochondria-specific metabolic reactions.

**Figure 3:**
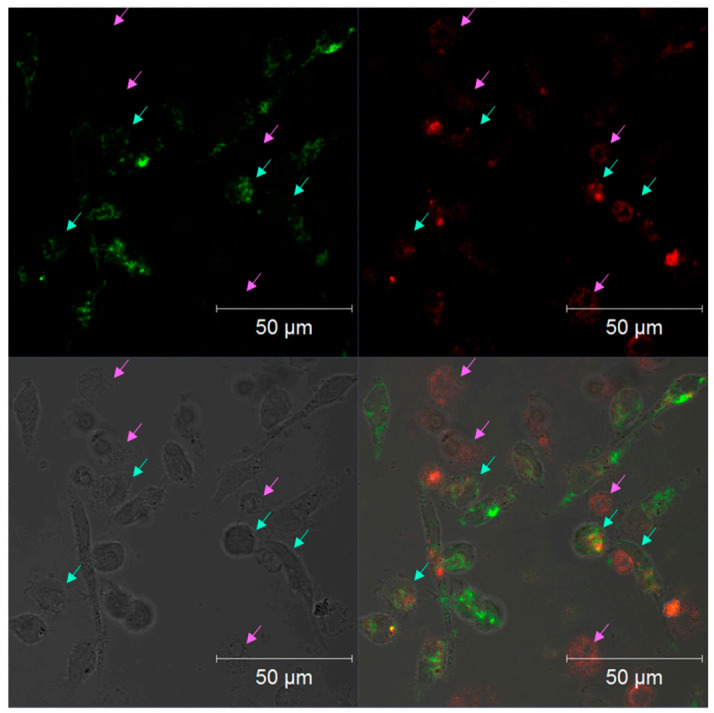
Confocal micrograph of CD14+ human monocytes stained with MitoTracker Orange CMTMRos showing protoporphyrin IX (PpIX) autofluorescence. MitoTracker Orange CMTMRos staining is shown in green, and PpIX autofluorescence is shown in red. MitoTracker-high/PpIX+ cells are marked with light-blue arrows. MitoTracker-low/PpIX+ cells are marked with purple arrows. Shown is a representative example of a field of view. Cells with different levels of mitochondrial membrane potential, revealed by the staining, are shown with arrows of different colors. Upper-left quadrant: MitoTracker fluorescence; upper-right quadrant: PpIX autofluorescence; lower-left quadrant: phase contrast; lower-right quadrant: combined image.

A confocal micrograph of monocytes incubated with 5-ALA is shown in Figure 3 and demonstrates two types of cells: MitoTracker-high/PpIX+ and MitoTracker-low/PpIX+ (Figure 3). In Figure 3, MitoTracker-high/PpIX+ are marked with blue arrows, while MitoTracker-low/PpIX+ cells are marked with purple arrows. Therefore, cells with impaired MMP can metabolize 5-ALA and accumulate PpIX. This indicates the partial preservation of mitochondrial function in MitoTracker-low cells. However, PpIX accumulation is also a sign of defective heme synthesis in the mitochondria.

It could be concluded that the primary culture of human monocytes contained cells with impaired MMP that were not, however, apoptotic and were capable of metabolizing 5-ALA to PpIX. We next tested whether there was between the association of the presence of such cells with atherosclerosis and the primary monocytes’ proinflammatory activation ability.

### 3.4. Association of MitoTracker-Low CD14+ Human Monocytes with the Proinflammatory Activation of Cells and Atherosclerosis Development

**Figure 4:**
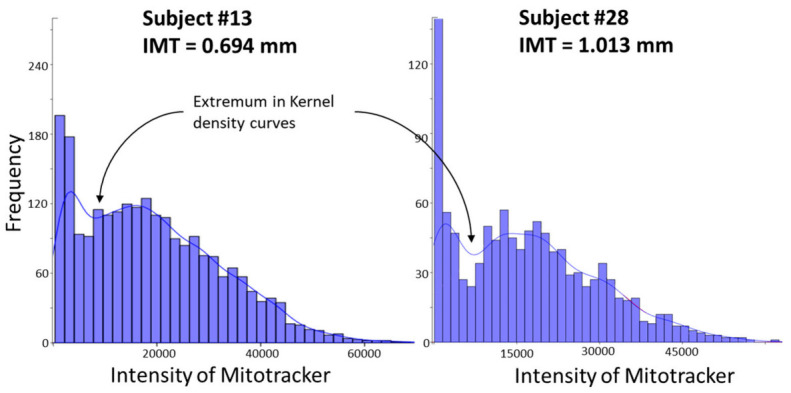
Bimodal distribution of the MitoTracker staining intensity of CD14+ human monocytes. Shown are the results for two study subjects with different intima-media thickness (IMT) values, corresponding to a normal (Subject 13) and an atherosclerotic (Subject 28) status. The extremum in the Kernel density curve used for the subsequent analysis of cell population on each histogram is shown with arrows.

To assess the proportion of MitoTracker-low CD14+ monocytes in the primary culture, we plotted the MitoTracker fluorescence intensity (Kernel density plot) for a cell population obtained from one patient (Figure 4). The distribution was found to be bimodal, and the extremum in the Kernel density curve was chosen for distinguishing the two cell subpopulations: MitoTracker-high and MitoTracker-low.

We next repeated the procedure and measured the proportion of MitoTracker-low cells in the primary monocyte cultures for each patient. A total of 36 subjects were analyzed, including 5 healthy donors aged <50 years, 7 healthy donors aged >50 years, and 23 patients with atherosclerosis. The results of the correlation analysis are presented in Table 2. We observed a negative correlation between the proportion of MitoTracker-low cells in the primary culture and the ability of cells to secrete TNF upon stimulation with LPS. There was no correlation between basal TNF secretion and the proportion of MitoTracker-low cells.

Therefore, an increased proportion of MitoTracker-low cells (cells with impaired MMP) in the monocyte primary culture was associated with a reduced LPS-induced proinflammatory activation ability. We next investigated the proportion of MitoTracker-low cells in different study participants and tested for a possible association with the presence of atherosclerotic changes.

**Figure 5:**
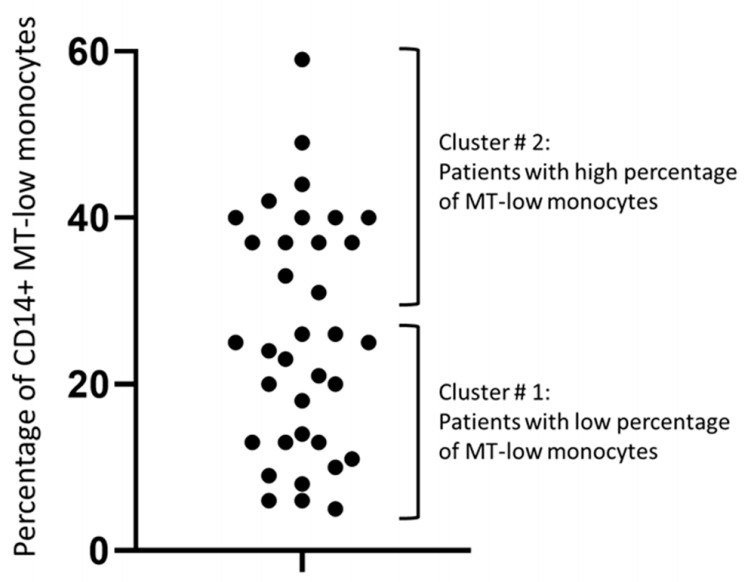
Bimodal distribution of patients by the proportion of MitoTracker-low monocytes allows distinguishing two clusters. MT, MitoTracker.

We found that the distribution of the proportion of MitoTracker-low monocytes among the study participants was not normal (*p* = 0.029) and had at least two modes (Figure 5). We therefore tested for a possible association between the proportion of MitoTracker-low cells and intima-media thickness (IMT) and the presence of atherosclerotic plaques. Subgroups were defined using k-means cluster analysis. Since the density curve of the proportion of MitoTracker-low monocytes was bimodal, k-means cluster analysis was performed for the two clusters.

We next analyzed the characteristics of patients from the two clusters with a low or high proportion of MitoTracker-low monocytes (hereafter Clusters 1 and 2, respectively). We found that patients from the MitoTracker-low Cluster 2 had a higher plaque score, and cells from these patients had a reduced proinflammatory activation ability, as assessed by LPS-induced TNF secretion (Table 3).

The obtained results allowed us to conclude that presence of MitoTracker-low monocytes was associated with atherosclerotic plaques and reduced proinflammatory activation of the immune cells.

## 4. Discussion

In this study, we revealed the presence of an elevated proportion of MitoTracker-low monocytes (with impaired MMP) in atherosclerotic patients. Morphologically, MitoTracker-low monocytes were not visibly different from the rest of the cells, as assessed by the cell size and granularity. Moreover, MitoTracker-low cells were able to metabolize 5-ALA and accumulate PpIX, indicative of the preservation of some of the mitochondrial functions. We assessed the proportion of MitoTracker-low cells in the primary monocyte culture obtained from each study participant and compared the results with other parameters, such as the monocytes’ proinflammatory activation ability, in response to the LPS and IMT of carotid arteries. We found that an elevated proportion of MitoTracker-low monocytes correlated with the presence of atherosclerotic plaques and reduced proinflammatory activation of monocytes.

A series of vital dyes sensitive to MMP in cultured cells are currently available, including MitoTracker^®^ Orange CMTMRos, JC1, and rhodamine 123. However, previous studies have revealed certain drawbacks of using JC and rhodamine 123. For instance, JC1 is not specific to the mitochondria, whereas the association of rhodamine 123 with mitochondria is reversible, and the dye is subject to rapid fading [11,12,13]. Therefore, we chose MitoTracker Orange CMTMRos, which is free from these drawbacks, as a potential-sensitive dye [14,15]. Additionally, all the mentioned dyes are used for studying mitochondrial function in different pathologies. For instance, the study of circulating monocytes from patients with type 2 diabetes using JC1 revealed that these cells had increased mitochondrial membrane potential, although the absolute intensity of aggregated and nonaggregated JC1 fluorescence in cells from patients was lower than in cells from control subjects, which could be explained by the reduced total volume of the mitochondria [16]. Other studies have demonstrated increased mitochondrial polarization in monocyte–thrombocyte aggregates from patients with rheumatoid arthritis and ischemic heart disease [17,18]. At the same time, HIV infection was shown to be associated with decreased MMP and altered morphology of the mitochondria in immune cells [19]. Reduced MMP was also demonstrated in sepsis [20]. Therefore, severe human pathologies can be associated with increased or reduced MMP.

In the current study, the proportion of MitoTracker-low monocytes was associated with the presence of atherosclerotic plaques in donor subjects and the reduced ability of monocytes to secrete TNF in response to LPS. Mitochondrial polarization, and hence their functional state, can influence the proinflammatory activity of immune cells, as demonstrated in a study of MMP in different human monocyte subpopulations. The authors showed that nonclassical monocytes presented reduced MMP, increased reactive oxygen species (ROS) production, and shortened telomeres compared to classical monocytes [21]. Another group studied monocyte-derived dendritic cells with impaired immune-stimulatory capacity. Such cells had decreased MMP and showed signs of being in a preapoptotic state [22]. Lugli et al.’s group demonstrated that upon apoptosis induction in vitro, immunocompetent cells could be classified into three phenotypes: normal, intermediate, and reduced MMP [23]. The latter had a reduced total mitochondrial volume and mtDNA content. Therefore, our results are in line with previous observations by different groups. At the cellular level, reduced MMP in immune cells was associated with preapoptotic or senescent states. At the organism level, mitochondrial depolarization was associated with various pathologies associated with mitochondrial dysfunction, such as HIV infection and sepsis.

Numerous studies summarized in a recent review have revealed changes in the metabolic signature of macrophages activated toward classical proinflammatory and alternative anti-inflammatory phenotypes [24]. Proinflammatory phenotypes are characterized by the preferential use of glycolysis for energy production, whereas alternatively activated macrophages rely more on oxidative phosphorylation. Furthermore, lipids and amino acids also differ in pro- and anti-inflammatory macrophages. It is safe to assume that such profound changes in metabolism involve the mitochondrial function of the cells and vice versa, and altered mitochondrial function due to the accumulation of mtDNA mutations may predispose the cells to proinflammatory activation. Further studies by our group will include the metabolic profiling of monocytes/macrophages with an intact affected mitochondrial function to clarify this question.

The obtained results allowed assuming that mitochondrial dysfunction in circulating monocytes can be linked to an impaired proinflammatory response and the appearance of atherosclerotic lesions. Our work has a number of limitations. The reduced intensity of MitoTracker staining did not allow for evaluating the possible change of the total mitochondrial volume. It was also not possible to define whether the subpopulations of MitoTracker-high and MitoTracker-low cells varied in terms of the abundance of classical and nonclassical monocytes. We did not evaluate other characteristics of mitochondrial dysfunction, such as ROS formation, p-ERK activation, and the presence of mtDNA mutations. These questions will be clarified in future studies. However, the present work contributes to our understanding of the role of mitochondrial dysfunction in the chronification of inflammation in human pathologies.

## 5. Conclusions

We revealed two distinct subpopulations of peripheral blood monocytes with normal or reduced MMP, as assessed by live staining with the potential-sensitive dye MitoTracker Orange CMTMRos. An increased proportion of cells with reduced MMP was associated with the reduced ability of monocytes to secrete LPS-induced TNF in primary culture and with the presence of atherosclerotic lesions in the donor subjects.

## Figures and Tables

**Table 1:** Baseline characteristics of the study participants.

Characteristics	Group 0: Atherosclerosis-Free Individuals (<0 y.o.) (*n* = 14)	Group 1: Atherosclerosis-Free Individuals (>50 y.o.) (*n* = 28)	Group 2: Subclinical Atherosclerosis (>50 y.o.) (*n* = 22)	*p*-Value * (Group 1 vs. 2)
Age, y	38 ± 2	59 ± 2	67 ± 2	0.002
Gender, % male (n)	35% (13)	29% (8)	41% (9)	0.377
BMI (kg/m^2^)	28 ± 1	26 ± 1	27 ± 1	0.261
IMT	0.61 ± 0.02	0.71 ± 0.02	0.84 ± 0.03	<<0.01
Plaque score	0	0.6 ± 0.1	2 ± 0.1	<<0.01
Plasma TChol, mmol/L	5.2 ± 0.2	5.8 ± 0.3	5.9 ± 0.3	0.765
Plasma Tg, mmol/L	1.2 ± 0.1	1.3 ± 0.1	1.6 ± 0.1	0.086
Plasma LDLc, mmol/L	3.2 ± 0.1	3.6 ± 0.1	3.6 ± 0.2	0.497
Plasma HDLc, mmol/L	1.4 ± 0.1	1.5 ± 0.1	1.4 ± 0.1	0.993

BMI, body mass index; HDLc, high-density lipoprotein cholesterol; IMT, intima-media thickness; LDLc, low-density lipoprotein cholesterol; TChol, total cholesterol; Tg, triglycerides; y.o., years old; * Independent samples *t*-test.

**Table 2:** Correlation analysis of MitoTracker staining intensity with cultured monocyte proinflammatory activation ability.

Vs.	Proportion of MitoTracker-Low Monocytes, Pearson Coefficients	*p*-Value
TNF secretion by nonstimulated monocytes, pg/mL	−0.151	0.444
TNF secretion by LPS-stimulated monocytes, pg/mL	−0.459	0.014 *

LPS, lipopolysaccharide; TNF, tumor necrosis factor. * statistically significant difference, bivariate Pearson correlation coefficients with a two-tailed test of significance.

**Table 3:** Comparative analysis of study subjects with a low or high proportion of MitoTracker-low monocytes.

	Cluster 1: Patients with Low Proportion of MT-Low Monocytes (*n* = 21)	Cluster 2: Patients with High Proportion of MT-Low Monocytes (*n* = 14)	*p*-Value
IMT, mm	0.74 ± 0.03	0.79 ± 0.04	0.306
Plaque score	0.9 ± 0.2	1.5 ± 0.2	0.045 *
TNF basal secretion, pg/mL	890 ± 110	760 ± 140	0.464
TNF LPS-induced secretion, pg/mL	3150 ± 340	2160 ± 240	0.027 *
Increasement in TNF secretion	2260 ± 270	1400 ± 180	0.015 *
Fold-change of TNF secretion	3.8 ± 0.3	3.3 ± 0.4	0.381

IMT, intima-media thickness; LPS, lipopolysaccharide; MT, MitoTracker; TNF, tumor necrosis factor. * statistically significant difference, independent samples *t*-test.

**Figure A1:**
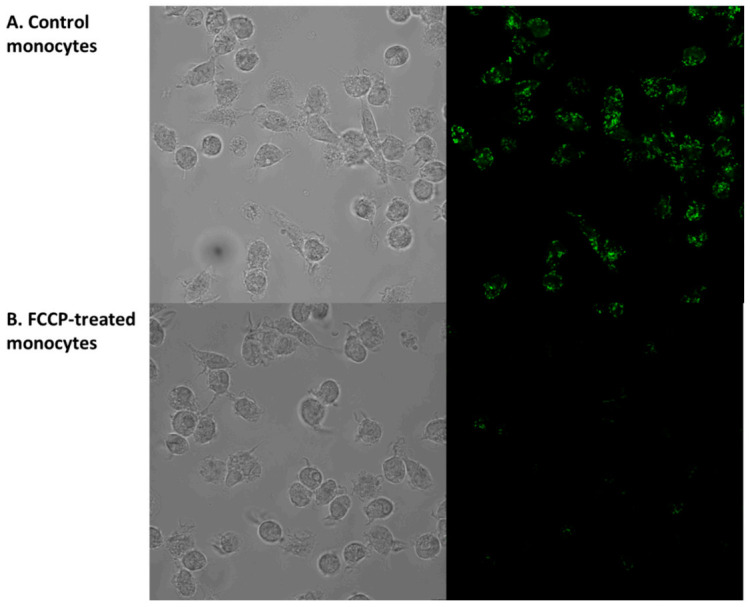
Monocytes were treated or not with 5 µM of carbonyl cyanide 4-(trifluoromethoxy)phenylhydrazone (FCCP) for 30 min to remove mitochondrial potential, stained with MitoTracker, and visualized as described above.

## Data Availability

Not applicable.

## Appendix A

Evaluation of the proinflammatory activation ability of CD14+ human monocytes in patients with asymptomatic (subclinical) atherosclerosis

Monocytes were isolated from the peripheral blood of healthy donors and patients with atherosclerosis. To assess CD14+ monocytes’ proinflammatory activation ability, cells were incubated with LPS, and the level of secreted TNF was measured (Table A1). LPS-induced TNF secretion was higher in monocytes isolated from atherosclerotic patients compared to healthy donors.

**Table A1:** Proinflammatory response of circulating monocytes.

Characteristics	Nonatherosclerotic Individuals (*n* = 28)	Subclinical Atherosclerosis (*n* = 22)	*p*-Value *
TNF secretion by nonstimulated monocytes, pg/mL	660 ± 70	740 ± 120	0.526
TNF secretion by LPS-stimulated monocytes, pg/mL	2640 ± 210	3120 ± 240	0.042
Increasement in TNF secretion, pg/mL	1910 ± 180	2200 ± 270	0.625
Fold-change of TNF secretion	1.36 ± 0.13	1.47 ± 0.12	0.349

* Independent samples *t*-test.

## Appendix B

Negative controls for MitoTracker staining.
